# Increased interleukin-17 production via a phosphoinositide 3-kinase/Akt and nuclear factor κB-dependent pathway in patients with rheumatoid arthritis

**DOI:** 10.1186/ar1470

**Published:** 2004-11-29

**Authors:** Kyoung-Woon Kim, Mi-La Cho, Mi-Kyung Park, Chong-Hyeon Yoon, Sung-Hwan Park, Sang-Heon Lee, Ho-Youn Kim

**Affiliations:** 1 Department of Medicine, Division of Rheumatology, The Center for Rheumatic Diseases, and The Rheumatism Research Center (RhRC), Catholic Research Institutes of Medical Sciences, Catholic University of Korea, Seoul, Korea

**Keywords:** interleukin-17, nuclear factor κB, PI3K/Akt pathway, peripheral blood mononuclear cells, rheumatoid arthritis

## Abstract

Inflammatory mediators have been recognized as being important in the pathogenesis of rheumatoid arthritis (RA). Interleukin (IL)-17 is an important regulator of immune and inflammatory responses, including the induction of proinflammatory cytokines and osteoclastic bone resorption. Evidence for the expression and proinflammatory activity of IL-17 has been demonstrated in RA synovium and in animal models of RA. Although some cytokines (IL-15 and IL-23) have been reported to regulate IL-17 production, the intracellular signaling pathways that regulate IL-17 production remain unknown. In the present study, we investigated the role of the phosphoinositide 3-kinase (PI3K)/Akt pathway in the regulation of IL-17 production in RA. Peripheral blood mononuclear cells (PBMC) from patients with RA (*n *= 24) were separated, then stimulated with various agents including anti-CD3, anti-CD28, phytohemagglutinin (PHA) and several inflammatory cytokines and chemokines. IL-17 levels were determined by sandwich enzyme-linked immunosorbent assay and reverse transcription–polymerase chain reaction. The production of IL-17 was significantly increased in cells treated with anti-CD3 antibody with or without anti-CD28 and PHA (*P *< 0.05). Among tested cytokines and chemokines, IL-15, monocyte chemoattractant protein-1 and IL-6 upregulated IL-17 production (*P *< 0.05), whereas tumor necrosis factor-α, IL-1β, IL-18 or transforming growth factor-β did not. IL-17 was also detected in the PBMC of patients with osteoarthritis, but their expression levels were much lower than those of RA PBMC. Anti-CD3 antibody activated the PI3K/Akt pathway; activation of this pathway resulted in a pronounced augmentation of nuclear factor κB (NF-κB) DNA-binding activity. IL-17 production by activated RA PBMC is completely or partly blocked in the presence of the NF-κB inhibitor pyrrolidine dithiocarbamate and the PI3K/Akt inhibitor wortmannin and LY294002, respectively. However, inhibition of activator protein-1 and extracellular signal-regulated kinase 1/2 did not affect IL-17 production. These results suggest that signal transduction pathways dependent on PI3K/Akt and NF-κB are involved in the overproduction of the key inflammatory cytokine IL-17 in RA.

## Introduction

Rheumatoid arthritis (RA) is characterized by infiltrations of macrophages and T cells into the joint, and synovial hyperplasia. Proinflammatory cytokines released from these cells are known to be important in the destruction of joints in RA [1]. The favorable clinical benefits obtained with inhibitors of tumor necrosis factor (TNF)-α) and interleukin (IL)-1 suggest that the blockade of key inflammatory cytokines has been the important issue in the development of new therapeutic applications [2].

A little over a decade ago, the primacy of T cells in the pathogenesis of autoimmune disease such as RA was undisputed because they are the largest cell population infiltrating the synovium. However, a series of studies demonstrated paucity of T cell-derived cytokines such as IL-2 and interferon-γ in the joints of RA, whereas macrophage and fibroblast cytokines including IL-1, IL-6, IL-15, IL-18 and TNF-α were abundant in rheumatoid synovium. This paradox has questioned the role of T cells in the pathogenesis of RA [3]. Because we have already demonstrated the enhanced proliferation of antigen specific T cells, especially to type II collagen, and the skewing of T helper type 1 (Th1) cytokines in RA [4], the role of T cells needs to be elucidated in different aspects.

IL-17 is one of the inflammatory cytokines secreted mainly by activated T cells, which can induce IL-6 and IL-8 by fibroblasts [5]. This cytokine is of interest for two major reasons: first, similarly to TNF-α and IL-1, IL-17 has proinflammatory properties; second, it is produced by T cells [6]. Recent observations demonstrated that IL-17 can also activate osteoclastic bone resorption by the induction of RANKL (receptor activator of nuclear factor κB [NF-κB] ligand), which is involved in bony erosion in RA [7]. It also stimulates the production of IL-6 and leukemia inhibitory factor by synoviocytes, and of prostaglandin E_2 _and nitric oxide by chondrocytes, and has the ability to differentiate and activate the dendritic cells [8-10]. Levels of IL-17 in synovial fluids were significantly higher in patients with RA than in patients with osteoarthritis (OA), and it was produced by CD4^+ ^T cells in the synovium [11,12].

IL-15, secreted from activated macrophages, has been reported to be an important trigger of IL-17 production in RA peripheral blood mononuclear cells (PBMC) by cyclosporine and steroid sensitive pathways [13]. Recently, Happel and colleagues also showed that IL-23 could be an efficient trigger of IL-17 production from both CD4^+ ^and CD8^+ ^T cells [14].

Although the contribution of IL-17 in joint inflammation in RA has been documented in earlier studies [12,15,16], the intracellular signal transduction pathway for IL-17 production remains uncertain. In the present study we used various stimuli to investigate IL-17 production in PBMC of patients with RA and its signaling transduction pathway.

We found that the intracellular signaling pathway involving phosphoinositide 3-kinase (PI3K)/Akt and NF-κB might be involved in the overproduction of the key inflammatory cytokine IL-17 in RA. These results might provide new insights into the pathogenesis of RA and future directions for new therapeutic strategies in RA.

## Materials and methods

### Patients

Informed consent was obtained from 24 patients (5 men and 19 women) with RA who fulfilled the 1987 revised criteria of the American College of Rheumatology (formerly the American Rheumatism Association) [17]. The age of the patients with RA was 50 ± 8 (mean ± SEM) years (range 23–71 years). All medications were stopped 48 hours before entry to the study. Comparisons were made with 14 patients with OA (3 men and 11 women) and with 14 healthy controls (3 men and 11 women) who had no rheumatic diseases. The mean ages of the patients with OA and the healthy controls were 50 ± 8 years (range 34–68 years) and 30 ± 6 years (range 24–57 years). Informed consent was obtained, and the protocol was approved by the Catholic University of Korea Human Research Ethics Committee.

### Reagents

Recombinant IL-17, IL-18, IL-15, monocyte chemoattractant protein-1 (MCP-1), macrophage inflammatory protein (MIP)-1α, MIP-1β, IL-6 and IL-8 were purchased from R & D systems (Minneapolis, MN, USA). Recombinant transforming growth factor (TGF)-β was purchased from Peprotech (London, UK). Recombinant TNF-α and IL-1 were purchased from Endogen Inc. (Cambridge, MA, USA). Cyclosporin A was provided by Sandos Ltd. (Basel, Switzerland). Phytohemagglutinin (PHA), pyrrolidine dithiocarbamate (PDTC), rapamycin, dexamethasone and curcumin were all obtained from the Sigma Chemical Co. (St Louis, MA, USA). Anti-CD3 monoclonal antibody and anti-CD28 monoclonal antibody were obtained from BD Biosciences (San Diego, CA, USA). LY294002, SB203580, FK506, wortmannin and PD98059 were obtained from Calbiochem (Schwalbach, Germany).

### Production of IL-17 by T cell receptor activation, cytokines or chemokines

PBMC were prepared from heparinized blood by Ficoll-Hypaque (SG1077) density-gradient centrifugation. Cell cultures were performed as described previously [18]. In brief, the cell suspensions were adjusted to a concentration of 10^6^/ml in RPMI 1640 medium supplemented with 10% fetal calf serum, 100 U/ml penicillin, 100 mg/ml streptomycin and 2 mM L-glutamine. Cell suspension (1 ml) was dispensed into 24-well multi-well plates (Nunc, Roskilde, Denmark), and incubated for 24 hours at 37°C in 5% CO_2_. Subsequently, various concentrations of cyclosporin A (10–500 ng/ml) were added to the medium and cells were incubated for 24 hours. To each well was added FK506, rapamycin, curcumin, PDTC, LY294002, SB203580, PD98059, dexamethasone or wortmannin. After incubation for 24 hours (unless otherwise stated), cell-free media were collected and stored at -20°C until assayed. All cultures were set up in triplicate, and results are expressed as means ± SEM.

### CD4^+ ^T-cell isolation by MACS

Anti-CD4 microbeads were used essentially as recommended by the manufacturer (Miltenyi) [19]. PBMC were resuspended in 80 μl of FBS staining buffer. Anti-CD4 microbeads (20 μl) were added and incubated for 15 min at 6–12°C. Saturating amounts of fluorochrome-conjugated antibodies were added for a further 10 min. Cells were diluted in 2.5 ml of FBS staining buffer, pelleted, resuspended in 500 μl and magnetically separated, usually on an AutoMACS magnet fitted with a MACS MS column. Flow-through and two 1 ml washes were collected as the negative fraction. Enriched cells were collected in two 0.5 ml aliquots from the column after removal from the magnet. Alternatively, cells stained with anti-CD4–phycoerythrin were washed, magnetically labeled with anti-phycoerythrin microbeads (20 μl added to 80 μl of cell suspension; 15 min, 6–12°C), and magnetically separated as described above. The purity of cells was assessed by flow cytometric analysis of stained cells on a FACS Vantage sorter. Most (more than 97%) of the isolated cells had the CD4 T cell marker.

### Enzyme-linked immunosorbent assay of IL-17

IL-17 in culture supernatants was measured by sandwich enzyme-linked immunosorbent assay as described previously [20]. In brief, a 96-well plate (Nunc) was coated with 4 μg/ml monoclonal antibodies against IL-17 (R & D Systems) at 4°C overnight. After blocking with phosphate-buffered saline/1% bovine serum albumin (BSA)/0.05% Tween 20 for 2 hours at room temperature (22–25°C), test samples and the standard recombinant IL-17 (R & D Systems) were added to the 96-well plate and incubated at room temperature for 2 hours. Plates were washed four times with phosphate-buffered saline/Tween 20, and then incubated with 500 ng/ml biotinylated mouse monoclonal antibodies against IL-17 (R & D Systems) for 2 hours at room temperature. After washing, streptavidin–alkaline phosphate–horseradish peroxidase conjugate (Sigma) was incubated for 2 hours, then washed again and incubated with 1 mg/ml *p*-nitrophenyl phosphate (Sigma) dissolved in diethanolamine (Sigma) to develop the color reaction. The reaction was stopped by the addition of 1 M NaOH and the optical density of each well was read at 405 nm. The lower limit of IL-17 detection was 10 pg/ml. Recombinant human IL-17 diluted in culture medium was used as a calibration standard, ranging from 10 to 2000 pg/ml. A standard curve was drawn by plotting optical density against the log of the concentration of recombinant cytokines, and used for determination of IL-17 in test samples.

### Quantification of IL-17 mRNA by semiquantitative reverse transcription–polymerase chain reaction

PBMC were incubated with various concentrations of anti-CD3 in the presence or absence of inhibitors (LY294002, PDTC). After 16 hours of incubation, mRNA was extracted with RNAzol B (Biotex Laboratories, Houston, TX, USA) in accordance with the manufacturer's instructions. Reverse transcription of 2 μg of total mRNA was performed at 42°C using the Superscript™ reverse transcription system (Takara, Shiga, Japan). PCR amplification of cDNA aliquots was performed by adding 2.5 mM dNTPs, 2.5 U of *Taq *DNA polymerase (Takara) and 0.25 μM of sense and antisense primers. The reaction was performed in PCR buffer (1.5 mM MgCl_2_, 50 mM KCl, 10 mM Tris-HCl, pH 8.3) in a total volume of 25 μl. The following sense and antisense primers for each molecules were used: IL-17 sense, 5'-ATG ACT CCT GGG AAG ACC TCA TTG-3'; IL-17 antisense, 5'-TTA GGC CAC ATG GTG GAC AAT CGG-3'; glyceraldehyde-3-phosphate dehydrogenase (GAPDH) sense, 5'-CGA TGC TGG GCG TGA GTA C-3'; GAPDH antisense, 5'-CGT TCA GCT CAG GGA TGA CC-3'. Reactions were processed in a DNA thermal cycler (Perkin-Elmer Cetus, Norwalk, CT, USA) through cycles for 30 s of denaturation at 94°C, 1 min of annealing at 56°C for GAPDH and IL-17, followed by 1 min of elongation at 72°C. PCR rounds were repeated for 25 cycles each for both GAPDH and IL-17; this was determined as falling within the exponential phase of amplification for each molecule. The level of mRNA expression was presented as a ratio of IL-17 PCR product over GAPDH product.

### Western blot analysis of Akt, phosphorylated Akt and IκB-α

PBMC were incubated with anti-CD3 (10 μg/ml) in the presence or absence of LY294002 (20 μM). After incubation for 1 hour, whole cell lysates were prepared from about 10^7 ^cells by homogenization in the lysis buffer, and centrifuged at 14,000 r.p.m. (19,000 *g*) for 15 min. Protein concentrations in the supernatants were determined with the Bradford method (Bio-Rad, Hercules, CA, USA). Protein samples were separated by 10% SDS–PAGE and transferred to a nitrocellulose membrane (Amersham Pharmacia Biotech, Uppsala, Sweden). For western hybridization, membrane was preincubated with 0.1% skimmed milk in TBS-T buffer (0.1% Tween 20 in Tris-buffered saline) at room temperature for 2 hours, then primary antibodies against Akt, phosphorylated Akt and IκB-α (Cell Signaling Technology Inc., Beverly, MA, USA), diluted 1:1000 in 5% BSA/TBS-T, were added and incubated overnight at 4°C. After washing four times with TBS-T, horseradish peroxidase-conjugated secondary antibodies were added and allowed to incubate for 1 hour at room temperature. After TBS-T washing, hybridized bands were detected with the enhanced chemiluminescence (ECL) detection kit and Hyperfilm-ECL reagents (Amersham Pharmacia).

### Gel mobility-shift assay of NF-κB binding site

Nuclear proteins were extracted from about 5 × 10^6 ^PBMC. Oligonucleotide probes encompassing the NF-κB binding site of the human IL-17 promoter (5'-ATG ACC TGG AAA TAC CCA AAA TTC-3') were generated by 5'-end labeling of the sense strand with [γ-^32^P]dATP (Amersham Pharmacia) and T4 polynucleotide kinase (TaKaRa). Unincorporated nucleotides were removed by NucTrap probe purification columns (Stratagene, La Jolla, CA, USA). Nuclear extracts (2 μg of protein) were incubated with radiolabeled DNA probes (10 ng; 100,000 c.p.m.) for 30 min at room temperature in 20 μl of binding buffer consisting of 20 mM Tris-HCl, pH 7.9, 50 mM KCl, 1 mM dithiothreitol, 0.5 mM EDTA, 5% glycerol, 1 mg/ml BSA, 0.2% Nonidet P40 and 50 ng/μl poly(dI-dC). Samples were subjected to electrophoresis on nondenaturing 5% polyacrylamide gels in 0.5 × Tris-borate-EDTA buffer (pH 8.0) at 100 V. Gels were dried under vacuum and exposed to Kodak X-OMAT film at -70°C with intensifying screens. Rabbit polyclonal antibodies against NF-κB subunits p50, p65 and c-Rel were from Santa Cruz Biotechnology (Santa Cruz, CA, USA).

### Cell viability (Trypan blue dye exclusion assay)

For cell viability assays, the trypan blue dye exclusion method was used to evaluate the potential of direct cytotoxic effect of inhibitors on cells. After incubation for 24 hours, the cells were harvested and the percentage cell viability was calculated with the formula 100 × (number of viable cells/number of both viable and dead cells) [21].

### Statistical analysis

Data are expressed as means ± SEM. Statistical analysis was performed with Student's *t*-test for matched pairs. *P *values less than 0.05 were considered significant.

## Results

### IL-17 production in PBMC from patients with RA, patients with OA and normal individuals

**Figure 1:**
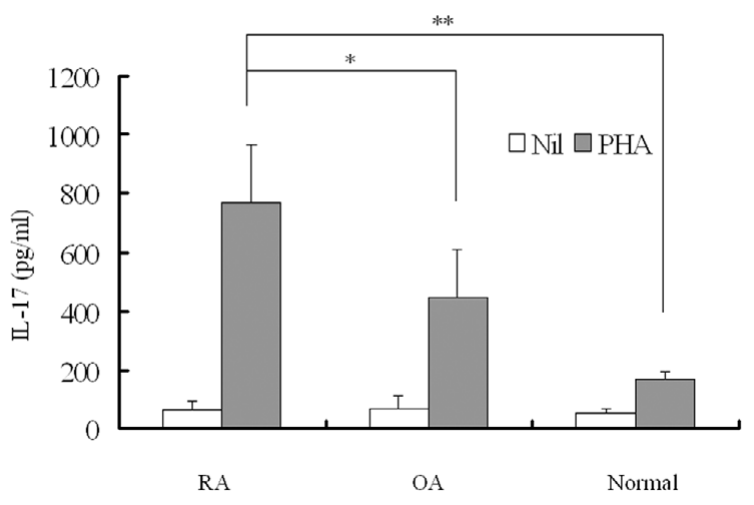
Levels of interleukin (IL)-17 production in peripheral blood mononuclear cells from patients with rheumatoid arthritis (RA; *n *= 24), patients with osteoarthritis (OA) (*n *= 14) and normal individuals (*n *= 14). Each peripheral blood mononuclear cell was stimulated for 24 hours with or without phytohemagglutinin (PHA; 5 μg/ml). IL-17 was measured in culture supernatants by sandwich enzyme-linked immunosorbent assay. Data are expressed as means and SEM. One representative result of five independent experiments is shown. Student's *t*-test was used to compare each group. *, *P *< 0.05; **, *P *< 0.001.

PBMC were separated and cultured with PHA (5 μg/ml) from patients with RA, patients with OA, and age-matched normal controls; IL-17 levels were then determined in the culture supernatants (Fig. 1). Although the amounts of basal IL-17 secretion were not different between RA, OA and normal controls (62 ± 31, 43 ± 19 and 43 ± 10 pg/ml, respectively), the IL-17 production stimulated by PHA was significantly higher in RA PBMC than in those from OA and controls (768 ± 295 versus 463 ± 211 pg/ml [*P *< 0.05] and 241 ± 29 pg/ml [*P *< 0.001]).

### Increased IL-17 production in PBMC of patients with RA by anti-CD3 and/or anti-CD28, and PHA

Because IL-17 was already known from earlier reports to be produced mainly by activated T cells, we investigated the effect of different concentrations of anti-CD3 (1, 5 and 10 μg/ml) as a T cell activation, which showed a dose-dependent increase in IL-17 levels (data not shown). On the basis of this, we chose 10 μg/ml as a stimulation concentration for anti-CD3. As shown in Table 1, anti-CD3 significantly upregulated IL-17 production up to 3.7-fold, and the combination of anti-CD28 and anti-CD3 produced more IL-17 (approximately 1.3-1.5-fold) than anti-CD3 alone. Furthermore, when incubated with T cell mitogens such as PHA, increased IL-17 production was more pronounced than with anti-CD3 and anti-CD28 (588 ± 85 versus 211 ± 1 pg/ml; *P *< 0.05).

### Regulation of IL-17 production in RA PBMC by inflammatory cytokines and chemokines

**Figure 2:**
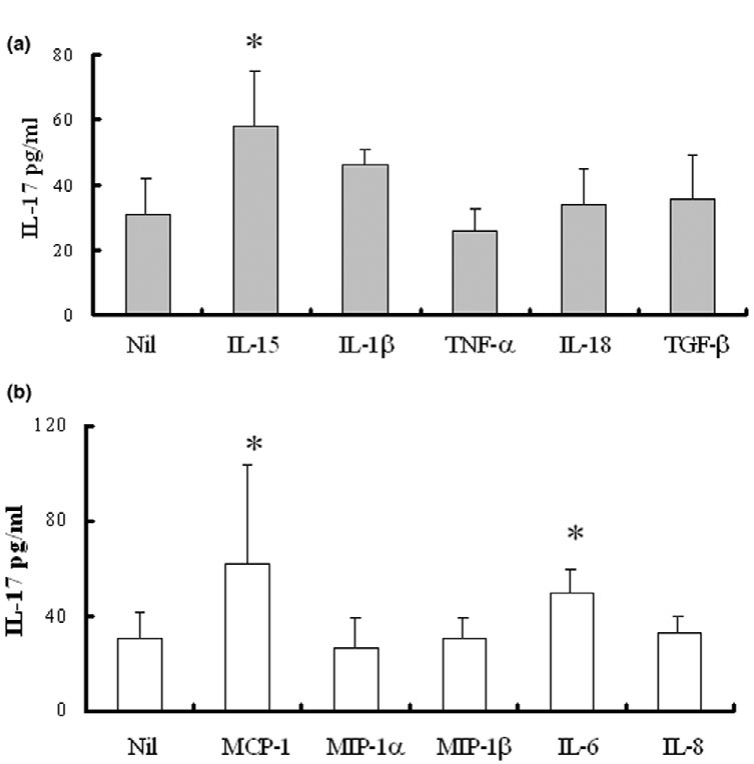
Production of interleukin (IL)-17 by peripheral blood mononuclear cells (PBMC) from patients with rheumatoid arthritis (RA). **(a) **Production of IL-17 by cytokine induction. PBMC from patients with RA were stimulated for 24 hours with IL-15 (10 ng/ml), IL-1β (10 ng/ml), tumor necrosis factor-α (TNF-α; 10 ng/ml), IL-18 (10 ng/ml) and transforming growth factor-β (TGF-β; 10 ng/ml). Levels of IL-17 were measured in culture supernatants by enzyme-linked immunosorbent assay. Each value represents the mean and SEM of three independent experiments. **(b) **Production of IL-17 by chemokine induction. PBMC were cultured in the presence of monocyte chemoattractant protein-1 (MCP-1; 10 ng/ml), macrophage inflammatory protein-1α (MIP-1α; 10 ng/ml), MIP-1β (10 ng/ml), IL-6 (10 ng/ml) and IL-8 (10 ng/ml). *, *P *< 0.05.

Because RA PBMC include several cell types in addition to T cells, some inflammatory cytokines released from macrophages and other lymphocytes might have affected the production of IL-17 from T cells. To evaluate the effects of inflammatory cytokines released by activated PBMC, we tested the effects of several cytokines and chemokines on IL-17 production. We detected an increase in IL-17 level after stimulation with IL-15 (10 ng/ml), whereas with IL-1β (10 ng/ml), TNF-α (10 ng/ml), IL-18 (10 ng/ml) or TGF-β (10 ng/ml) the levels in IL-17 were unchanged (Fig. 2a). When treated with MCP-1 (10 ng/ml) or IL-6 (10 ng/ml), significant upregulations of IL-17 proteins were observed (62 ± 42 and 50 ± 10 versus 31 ± 11 pg/ml, respectively; *P *< 0.05), whereas none was observed with IL-8 (10 ng/ml), MIP-1α (10 ng/ml) or MIP-1β (10 ng/ml) (Fig. 2b).

### Inhibition of IL-17 production by signal transduction inhibitors and anti-rheumatic drugs

**Figure 3:**
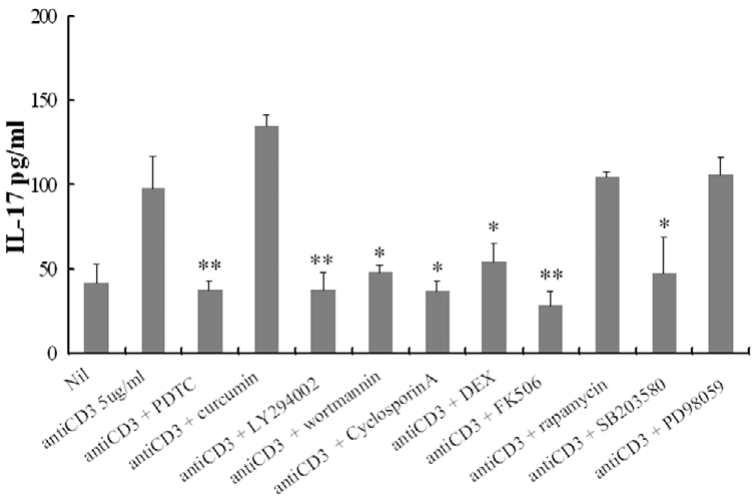
Effects of protein kinase inhibitors and anti-rheumatic drug on anti-CD3 triggered interleukin (IL)-17 production by peripheral blood mononuclear cells (PBMC) from patients with rheumatoid arthritis. PBMC pretreated for 1 hour with pyrrolidine dithiocarbamate (PDTC; 300 μM), curcumin (10 μM), LY294002 (20 μM), wortmannin (200 nM), Cyclosporin A (500 ng/ml), dexamethasone (DEX; 100 nM), FK506 (100 ng/ml), rapamycin (10 ng/ml), SB203580 (10 nM) or PD98059 (20 μM) in combination with anti-CD3 antibody (5 μg/ml). Culture supernatant was assayed for IL-17 as described in the Materials and methods section. Each value represents the mean and SEM of three independent experiments. *, *P *< 0.05; **, *P *< 0.005.

Having observed the increased IL-17 production in RA PBMC, it was important to know which signal transduction pathways were involved. As illustrated in Fig. 3, an significant decrease in anti-CD3-induced IL-17 production was observed when co-incubated with NF-κB inhibitor, PDTC and dexamethasone in comparison with anti-CD3 alone (38 ± 5 and 54 ± 11 versus 98 ± 19 pg/ml, respectively; *P *< 0.05).

LY294002 and wortmannin, as an inhibitor of PI3K, also markedly inhibited the anti-CD3-induced IL-17 production in RA PBMC (98 ± 19 versus 38 ± 10 pg/ml [*P *< 0.005] and 48 ± 4 pg/ml [*P *< 0.05], respectively).

**Figure 4:**
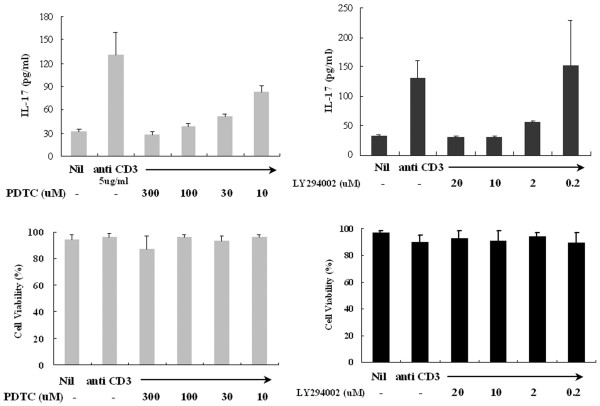
Dose-dependent effects of LY294002 or pyrrolidine dithiocarbamate (PDTC) in peripheral blood mononuclear cells (PBMC) from patients with rheumatoid arthritis (RA). **(a) **Effect of inhibitors on interleukin (IL)-17 release by anti-CD3-stimulated PBMC from patients with RA. **(b) **Effects of LY294002 or PDTC on PBMC viability. PBMC were cultured at a concentration of 2 × 10^5 ^cells per well with medium, anti-CD3, anti-CD3 and LY294002 or PDTC under the conditions described in the Materials and methods section. After 24 hours of treatment, cell viability was assessed by the trypan blue dye exclusion method and expressed as a percentage with the formula 100 × (number of viable cells/number of both viable and dead cells).

The calcineurin inhibitors cyclosporin A and FK506 also downregulated the IL-17 secretion as well as the mitogen-activated protein kinase (MAPK) p38 inhibitor SB203580 did, whereas rapamycin and PD98059 had no effect on IL-17 levels (Fig. 3). To evaluate the possibility of non-specific inhibition by the drug at high concentrations, we observed the dose response of PDTC and LY294002 for the inhibition of IL-17 production in PBMC. There were dose-dependent inhibitions of IL-17 production with chemical inhibitors (Fig. 4a). The other inhibitors in addition to PDTC and LY294002 showed the same pattern of inhibition. Cytotoxic effects on PBMC by the chemical inhibitors at experimental concentrations were not observed (Fig. 4b).

### IL-17 mRNA expression in RA PBMC

**Figure 5:**
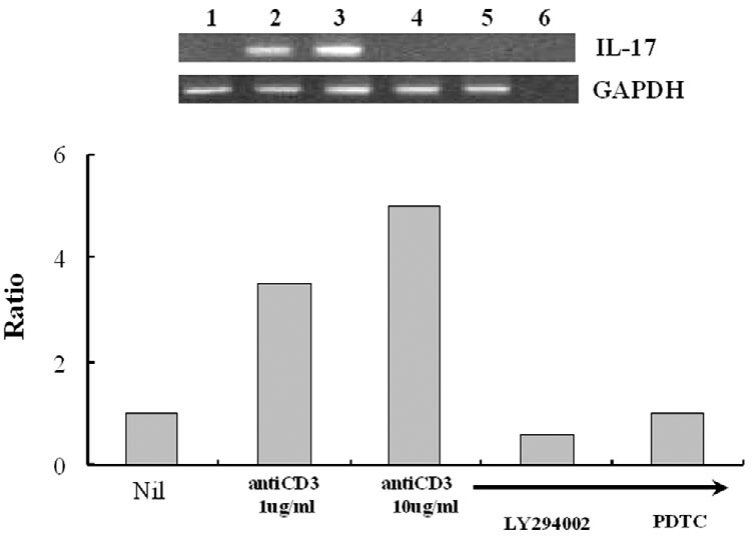
Effects of LY294002 or pyrrolidine dithiocarbamate (PDTC) on anti-CD3 antibody-triggered interleukin (IL)-17 mRNA expression by peripheral blood mononuclear cells (PBMC) from patients with rheumatoid arthritis. PBMC were cultured with medium only (lane 1), anti-CD3 antibody (1 μg/ml; lane 2), anti-CD3 antibody (10 μg/ml; lane 3), anti-CD3 antibody (10 μg/ml) plus LY294002 (20 μM; lane 4) or anti-CD3 antibody (10 μg/ml) plus PDTC (300 μM; lane 5) for 12 hours; lane 6 shows a negative control. Total RNA (2 μg) was used for cDNA synthesis in a volume of 20 μl; 1 μl of the synthesized cDNA was used for reverse transcription–polymerase chain reaction as described. PCR reaction product (25 μl) was separated on an agarose gel containing ethidium bromide. The relative intensities of the bands were revealed under UV radiation.

To see whether enhanced IL-17 production could be regulated at a transcriptional level, semi-quantatitive reverse transcription–polymerase chain reaction was performed. We observed a dose-dependent increase in IL-17 mRNA transcripts after stimulation with anti-CD3; this was inhibited by the PI3K inhibitor LY294002 and by the NF-κB inhibitor PDTC (Fig. 5).

### Activation of PI3K/Akt signal transduction pathway on IL-17 production by anti-CD3

**Figure 6:**
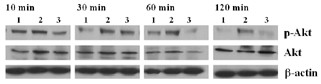
Activation of phosphorylated Akt after interleukin (IL)-17 induction by anti-CD3 antibody, and its inhibition by LY294002. Peripheral blood mononuclear cells were cultured with medium only (lane 1), anti-CD3 antibody (10 μg/ml; lane 2) or anti-CD3 antibody (10 μg/ml) plus LY294002 (20 μM; lane 3) for 10–120 min. Cell lysates were analyzed for Akt activation by western blot analysis of total and Ser473-phosphorylated Akt (P-Akt) using specific antibodies. Levels of phosphorylated Akt were compared at each time point, after normalization to Akt and β-actin in the same sample. A representative example of three separate experiments is shown.

To determine downstream effector molecules of the PI3K pathway, we evaluated the activation of Akt by western blotting. As shown in Fig. 6, at 10 min of incubation with anti-CD3 (10 μg/ml) or LY294002 (20 μM), no difference in the amounts of phosphorylated Akt was observed. However, after 30 min of incubation, phosphorylated Akt increased (lane 2), and the effect of inhibition by LY294002 (lane 3) reached a peak at 60 min, lasting to 120–240 min. In contrast, non-phosphorylated Akt and β-actin remained unchanged regardless of incubation time. PHA, concanavalin A and IL-15 also demonstrated the same effect on phosphorylated Akt as shown with anti-CD3, which was an inhibition by wortmannin and PDTC as well as by LY294002 (data not shown).

### Activation of the NF-κB and activator protein-1 (AP-1) pathway in the IL-17 promoter region

**Figure 7:**
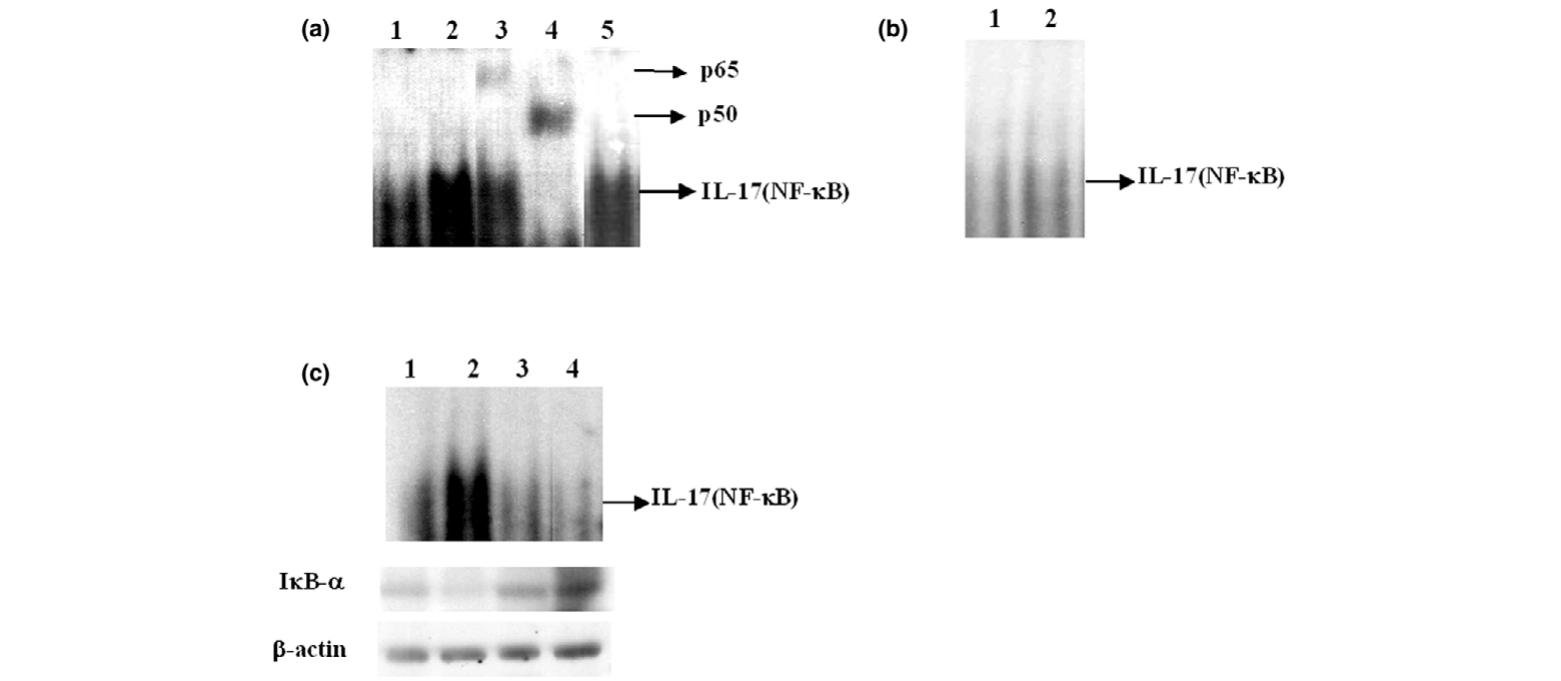
Effects of anti-CD3 plus anti-CD28 on NF-κB complex in nuclear extracts of rheumatoid arthritis (RA) peripheral blood mononuclear cells (PBMC) and normal PBMC. **(a) **NF-κB activity in the absence (lane 1) or presence (lane 2) of anti-CD3 plus anti-CD28 antibody; supershift assay of NF-κB site with antibodies against p65 (lane 3), p50 (lane 4) and c-Rel (lane 5). PBMC from patients with RA were stimulated with anti-CD3 plus anti-CD28 and were used for the supershift assay. **(b) **NF-κB activity in the absence (lane 1) or presence (lane 2) of anti-CD3 antibody plus anti-CD28 in normal PBMC. **(c) **NF-κB activity in the absence (lane 1) or presence (lane 2) of anti-CD3, anti-CD3 plus LY294002 (lane 3) or anti-CD3 plus pyrrolidine dithiocarbamate (PDTC; lane 4). Arrows denote a labeled oligonucleotide band shifted after NF-κB binding. The lower panel shows an immunoblot for IκB-α and actin at the same time.

To investigate further the intracellular signaling pathway activated by anti-CD3 plus anti-CD28, concanavalin A, PHA and IL-15, and responsible for inducing IL-17 expression, we performed an electrophoretic mobility-shift assay (EMSA) of NF-κB recognition sites in the promoters of IL-17. As shown in Fig. 7a, nuclear extracts from RA PBMC stimulated with anti-CD3 plus anti-CD28 (lane 2) demonstrated increased binding of NF-κB to IL-17 promoters in comparison with that of controls (lane 1). A supershift assay demonstrated shifted bands in p65 and p50 (lanes 3 and 4) not in c-Rel (lane 5). In normal PBMC the same pattern was observed, but the degree of NF-κB activation by anti-CD3 plus anti-CD28 was less intense than that in RA PBMC (Fig. 7b). To confirm the link between PI3K activity and NF-κB, we performed EMSA to determine the NF-κB binding activity after treatment with both LY294002 and PDTC. Both agents block NF-κB DNA-binding activity in the IL-17 promoter (Fig. 7c). Western blotting for IκB-α showed inhibition of degradation of IκB-α by LY294002 and PDTC at the same time (Fig. 7c). In contrast, the AP-1 pathway was not activated by stimulation with anti-CD3 plus anti-CD28 (data not shown), demonstrating that NF-κB is the main intracellular signaling pathway in IL-17 production by activated PBMC from patients with RA.

## Discussion

IL-17 was first described as a T cell product with proinflammatory properties [5,22]. RA is characterized by hyperplasia of synovial lining cells and an intense infiltration by mononuclear cells [23]. Proinflammatory cytokines such as IL-1 and TNF-α are abundant in rheumatoid synovium, whereas the T cell-derived cytokines, especially IL-4 and interferon-γ, have often proved difficult to detect in RA synovium [24]. Although T cells may have a role in the augmentation of rheumatoid synovial inflammation, the lack of T cell-derived cytokines has limited its importance. In this respect, IL-17 is appealing because it has been described as a T cell-derived cytokine with proinflammatory properties.

In our studies, we tried to evaluate how IL-17 production is regulated in RA PBMC, and which signaling pathway it used. Levels of IL-17 were found to be higher in RA synovial fluid than in OA synovial fluid [15]. However, there are few data available on the agents that stimulate IL-17 production in RA, although the highest level of IL-17 production can be achieved by anti-CD3/anti-CD28 stimulation in healthy individuals [25]. In our experiments, PHA as mitogens, as well as anti-CD3/anti-CD28 for signaling through the T cell receptor, increased IL-17 production from RA PBMC in a dose-dependent manner. We found, by a cell proliferation assay (data not shown), that this upregulation of IL-17 might be due to increased cellular activity rather than to cellular proliferation.

IL-17 is produced mainly by activated CD4^+ ^T cells, especially for Th1/Th0 cells, not the Th2 phenotype [26]. However, it can also be produced by CD8^+ ^T cells via an IL-23 triggering mechanism in Gram-negative pulmonary infection [14]. In addition, IL-17 production was significantly augmented by T cells recognizing type II collagen in a collagen-induced arthritis model [27]. A complex interaction between cells in inflamed RA joints might produce a variety of proinflammatory cytokines and chemokines, which also activate other cells in the joints. For example, IL-17 stimulates rheumatoid synoviocytes to secrete several cytokines such as IL-6, IL-8 and tumor necrosis factor-stimulated gene 6 as well as prostaglandin E_2 _*in vitro *[12,28,29]. There are as yet few data available on the agents that stimulate IL-17 production in RA, although some cytokines (IL-15 and IL-23) have been known to regulate IL-17 production [13,14]. We therefore investigated the *in vitro *production of IL-17 in RA PBMC responding to a variety of cytokines/chemokines and mitogens as well as T cell receptor (TCR) ligation using anti-CD3/anti-CD28. Our studies demonstrated that IL-15 and MCP-1 as well as TCR ligation significantly increased the production of IL-17 in RA PBMC. Adding IL-15 or MCP-1 to TCR ligation augmented IL-17 production more markedly. In contrast, IL-1 and TNF-α, which are known to have proinflammatory properties and to be increased in RA joints, did not affect IL-17 production. Our data were consistent with a recent report that IL-15 triggered *in vitro *IL-17 production in PBMC, but TNF-α did not do so [13]. Although there were no data that MCP-1 directly induces T cell activation, it might exert effects indirectly on T cells through the activation of monocytes/macrophages in PBMC cultures. As reported for normal individuals [25], T cell activation through anti-CD3/anti-CD28 also increases IL-17 induction in RA PBMC.

Although the signaling pathway for the induction of cytokines/chemokines by IL-17 has been documented widely [8,30,31], no data have been available on how IL-17 production can be regulated by certain signaling pathways. By using signal transduction inhibitors, we therefore examined which signaling pathway was mainly involved in the induction of IL-17 in RA PBMC.

We identified that anti-CD3-induced IL-17 production in RA PBMC was significantly hampered by the PI3K inhibitor LY294002 and the NF-κB inhibitor PDTC to comparable levels of basal production without stimulation. We also found that anti-CD3-induced IL-17 production was downregulated by the addition of SB203580, a p38 MAPK inhibitor. It is interesting that a series of evidence supports crosstalk between NF-κB and p38. In myocytes, IκB kinase-β is activated by p38 [32], and the activated p38 can stimulate NF-κB by a mechanism involving histone acetylase p300/CREB-binding protein [33]. Our results revealed that p38 MAPK activation was not affected by LY294002, whereas NF-κB binding activity was decreased by LY294002, which provided the evidence for a p38 MAPK pathway independent of PI3K activation. The direct relationship between p38 and NF-κB for IL-17 production needs to be studied in future experiments.

The search for a downstream pathway of PI3K seemed to have a maximal response of Akt activation at 1 hour and a gradual loss of activity at 2 hours. The fact that Akt is phosphorylated upon anti-CD3 stimulation suggests the possible involvement of PI3K in the induction of IL-17 in RA. In view of the fact that NF-κB was also activated by anti-CD3/anti-CD28, IL-15 or mitogens in our experiments, it is most likely that the NF-κB pathway is also actively involved in the induction of IL-17 in RA PBMC. In contrast, the AP-1 signal transduction pathway, another important signaling pathway for cytokines/chemokines, was not activated in our experiments (data not shown). Although PI3K and its downstream kinase Akt in association with NF-κB have been reported to deliver activating signals in many cell types, the data on the signal inducing IL-17 are lacking. Our data clearly demonstrated that PI3K/Akt and resultant NF-κB activation could be an important arbitrator of the upregulation of IL-17 in RA, on the basis of our experiments showing simultaneous blocking of NF-κB binding activity in the IL-17 promoter by PDTC and LY294002. Considering its proinflammatory activities and successful induction of anti-IL-17 for ameliorating arthritis in animal models [2,6,34-36], understanding the IL-17 signaling pathway is an important element of developing new targeted therapies in RA.

## Conclusions

We have detected a more pronounced production of IL-17 from RA PBMC in response to IL-15 and MCP-1 as well as stimulation by anti-CD3/anti-CD28. We have also shown that upregulation of IL-17 by activated T cells in patients with RA could be the result of activation via the PI3K/Akt pathway with resultant NF-κB activation. Our data provide insights into cellular mechanisms of the regulation of IL-17 production in RA, and highlight the role of T cells, which has hitherto been neglected in RA pathogenesis. Together with recent data on the successful introduction of anti-IL-17 in RA, our results have added information for the future molecular targeting of new therapeutic applications in RA.

## Abbreviations

AP-1, activator protein-1; BSA = bovine serum albumin; EMSA = electrophoretic mobility-shift assay; GAPDH = glyceraldehyde-3-phosphate dehydrogenase; IL = interleukin; MAPK = mitogen-activated protein kinase; MCP-1 = monocyte chemoattractant protein-1; MIP = macrophage inflammatory protein; NF-κB = nuclear factor κB; OA = osteoarthritis; PBMC = peripheral blood mononuclear cells; PDTC = pyrrolidine dithiocarbamate; PHA = phytohemagglutinin; PI3K = phosphoinositide 3-kinase; RA = rheumatoid arthritis; TGF = transforming growth factor; Th = T helper; TNF = tumor necrosis factor.

## Competing interests

The author(s) declare that they have no competing interests.

## Authors' contributions

KWK performed the cellular immune response studies and participated in the immunoassays. MLC participated in the design of the study and performed the statistical analysis. MKP participated in the isolation of the cells. CHY drafted the manuscript. SHP participated in the molecular biology and in the PCR. SHL conceived the study, participated in its design and coordination and helped to draft the manuscript. HYK helped to draft the manuscript. All authors read and approved the final manuscript.

## Figures and Tables

**Table 1:** Production of interleukin-17 in response to anti-CD3 and mitogens by peripheral blood mononuclear cells and T cells from patients with rheumatoid arthritis

RA cells	Stimulation	Interleukin-17 (pg/ml)
PBMC	None	42 ± 11
	Anti-CD3	155 ± 24
	Anti-CD3 + anti-CD28	211 ± 1
	PHA	588 ± 85
T cells	None	30 ± 10
	Anti-CD3	94 ± 41
	PHA	122 ± 73

Rheumatoid arthritis (RA) peripheral blood mononuclear cells (PBMC) were stimulated for 24 hours with anti-CD3 (10 μg/ml) plus anti-CD28 antibody (1 μg/ml), phytohemagglutinin (PHA; 5 μg/ml), or none of these (medium only). RA T cells were stimulated for 24 hours with anti-CD3 (10 μg/ml) and PHA (5 μg/ml). The levels of interleukin-17 were measured in culture supernatants by enzyme-linked immunosorbent assay. Results are means ± SEM of three independent experiments.
